# Long-term moderate alcohol consumption does not exacerbate age-related cognitive decline in healthy, community-dwelling older adults

**DOI:** 10.3389/fnagi.2014.00341

**Published:** 2015-01-05

**Authors:** Malaak N. Moussa, Sean L. Simpson, Rhiannon E. Mayhugh, Michelle E. Grata, Jonathan H. Burdette, Linda J. Porrino, Paul J. Laurienti

**Affiliations:** ^1^Laboratory for Complex Brain Networks, Department of Radiology, Wake Forest School of MedicineWinston-Salem, NC, USA; ^2^Neuroscience Program, Wake Forest School of MedicineWinston-Salem, NC, USA; ^3^Department of Biostatistical Sciences, Wake Forest School of MedicineWinston-Salem, NC, USA; ^4^MD Program, Wake Forest School of MedicineWinston-Salem, NC, USA; ^5^Department of Radiology, Wake Forest School of MedicineWinston-Salem, NC, USA; ^6^Department of Physiology and Pharmacology, Wake Forest School of MedicineWinston-Salem, NC, USA

**Keywords:** alcohol, moderate, chronic, aging, cognition

## Abstract

Recent census data has found that roughly 40% of adults 65 years and older not only consume alcohol but also drink more of it than previous generations. Older drinkers are more vulnerable than younger counterparts to the psychoactive effects of alcohol due to natural biological changes that occur with aging. This study was specifically designed to measure the effect of long-term moderate alcohol consumption on cognitive health in older adult drinkers. An extensive battery of validated tests commonly used in aging and substance use literature was used to measure performance in specific cognitive domains, including working memory and attention. An age (young, old) ^*^ alcohol consumption (light, moderate) factorial study design was used to evaluate the main effects of age and alcohol consumption on cognitive performance. The focus of the study was then limited to light and moderate older drinkers, and whether or not long-term moderate alcohol consumption exacerbated age-related cognitive decline. No evidence was found to support the idea that long-term moderate alcohol consumption in older adults exacerbates age-related cognitive decline. Findings were specific to healthy community dwelling social drinkers in older age and they should not be generalized to individuals with other consumption patterns, like heavy drinkers, binge drinkers or ex-drinkers.

## Introduction

Alcohol consumption among older adults is on the rise in the United States. To illustrate this point, the cumulative probability of exposure to alcohol before the age of 100 years old was confined to approximately 50% of the US population between the years of 1894 and 1937, and in 1974 it rose to 75% by the age of 30 years old (USDA and USDHHS, 2010; Wang and Andrade, 2013). The most recent National Survey on Drug Use and Health conducted in 2012 found that approximately 40% of the estimated 43 million older adults in the US consumed at least one alcoholic drink within the last month and that the majority of these individuals were neither heavy drinkers nor binge drinkers (SAMHSA, 2013). In fact, findings from the National Longitudinal Alcohol Epidemiologic Survey (NLAES) (NIAAA, 1998) and the National Epidemiologic Survey on Alcohol and Alcohol Related Conditions (NESARC) (Fryar et al., 2006; NIAAA, 2006) show that the majority of older American drinkers consume light (≤3 drinks/week) to moderate (7–14 drinks/week) amounts of alcohol. Potential reasons for an overall increase in moderate alcohol consumption may stem from the belief that exposure to moderate levels of alcohol is associated with psychological (Peele and Brodsky, 2000) and physiological (Marchi et al., 2014) benefits as well as a lower risk of developing dementia syndromes (Panza et al., 2009, 2012).

In today's world, older adults enjoy longer lives and rely on their cognitive abilities for many years prior to the debilitating losses in brain function associated with dementia (Plassman et al., 2007; WHO, 2012). Older adults, however, are still subject to significant biological changes that occur with normal aging and that make them particularly vulnerable to alcohol exposure. For example, increases in the proportion of fat to water and decreases in the rate of liver metabolism expose older adults to higher blood alcohol content (BAC) levels relative to young adults with similar overall body mass (Vestal et al., 1977). Ethanol, which acts directly on the central nervous system, has psychoactive properties that are known to significantly affect behaviors like attention, working and short-term memory (Guillot et al., 2010; Dry et al., 2012; Magrys and Olmstead, 2014). These neurologic effects can be transient and reversible in the case of acute alcohol exposure (Gilbertson et al., 2009; Sklar et al., 2012). On the other hand, problematic drinking habits like those practiced by older heavy drinkers and alcoholics have been firmly associated with severe cognitive deficits that can persist even after years of abstinence (Thomas and Rockwood, 2001; Peters et al., 2008).

It is not clear, however, whether or not long-term moderate (i.e., social) drinking affects age-related cognitive decline. Research addressing this topic is primarily epidemiologic in nature and dominated by analyses that use secondary outcome measures from large-scale clinical trials, like the Framingham Heart Study (Elias et al., 1999), the Seattle Longitudinal Study (Zanjani et al., 2013) and the Women's Health Initiative (Espeland et al., 2005, 2006). These and similar trials collected summary measures related to alcohol consumption and overall cognitive health but were not specifically designed to measure the effect of moderate alcohol consumption on age-related cognitive decline as the primary outcome. See reviews for more detail (Solfrizzi et al., 2007; Peters et al., 2008; Panza et al., 2009, 2012; Kim et al., 2012).

This study was specifically designed to determine the effect of long-term moderate alcohol consumption on age-related cognitive decline in healthy community dwelling adults. A collection of validated tests commonly used in aging as well as substance use literature was used to evaluate attention, working memory, short-term memory, processing speed, planning, rule learning, and impulse control. The first study hypothesis was that younger drinkers would outperform older drinkers in all the cognitive domains tested. Heavy alcohol use in older age has been linked to severe deficits in cognitive health (Moriyama et al., 2006; Green et al., 2010) and because of this it was thought that the effect of alcohol consumption level on cognition would follow a continuum. Therefore, the second study hypothesis was that light older drinkers would outperform moderate older drinkers on all the cognitive domains tested. At present, there is no curative treatment or therapeutic approach to reversing age-related declines in cognitive function. Understanding whether or not older adults are more vulnerable to such declines as a result of long-term moderate alcohol consumption will help a growing segment of the older American population make informed decisions about their cognitive health.

## Methods

Our main study question focused on the effect of moderate alcohol consumption on cognitive function in older adults, and to best address this question we adopted an age (young, old) ^*^ alcohol consumption (light, moderate) factorial study design. All participants were residents of Winston-Salem, North Carolina, and were recruited via local advertisements (physical flyers and internet) and by word-of-mouth. The Institutional Review Board of Wake Forest School of Medicine approved the study (IRB # 19961) and all participants gave written informed consent.

### Study population

A total of 63 individuals were enrolled in the study, and of these individuals 22 were younger adults (24–35 years old) and 41 were older adults (65–80 years old). The final study groups were as follows: 11 light young, 11 moderate young, 20 light old, and 21 moderate old drinkers. Census findings from the NLAES and the NESARC were used to help define light and moderate alcohol consumption criteria. Criteria were also defined such that light and moderate alcohol intake did not overlap. Light alcohol consumption was defined to be 1–8 drinks per month and did not exceed two drinks per week. Moderate alcohol consumption was defined as 7–21 drinks per week and did not exceed three drinks per day. Enrollment in the study was dependent on the self-report number of drinks per week as measured by the Time Line Follow Back (TLFB) questionnaire (Sobell and Sobell, 1992). Daily alcohol intake over 3 months was measured and only adults who reported maintaining either a light or moderate alcohol consumption pattern for at least 3 years were included.

### Exclusion criteria

#### Alcohol and other substance use

Participants were excluded if they reported more than one binge in the last 3 months (≥5 drinks for men and ≥4 drinks for women in less than 2 h) (NIAAA, 2004). Disqualification from the study also included a >0.00 alcohol reading as measured by an Intoxilyzer S-D5 breath alcohol screen (www.alcoholtest.com), or a positive test for illicit substance use as measured by an Alere iCassette 6 – panel drug screen at the start of any study visit (cocaine, THC, opiates, amphetamines, methamphetamines or benzodiazepines, www.alere.com). Use of illicit substances within the last 3 months was also grounds for disqualification. Subjects with a history of alcohol or drug abuse/dependence and/or current (within the last 6 months) Axis I disorders as determined by the Structured Interview for DSM Disorders (SCID) were also excluded (Fist et al., 2002). Individuals who consumed greater than 500 mg of caffeine per day were not included in this study so as to avoid the effects of excessive caffeine exposure or caffeine withdrawal on cognitive performance. Also, individuals who smoked ≤30 cigarettes (i.e., individuals defined by the World Health Organization as non-smokers – heavy smokers) were allowed to participate in the study so that during recruitment any potential confounding difference in cigarette smoking between light and moderate drinkers could be avoided.

#### Cognitive

Participants at high risk for dementia as measured by the Modified Mini Mental State Exam (3MSE score ≤ 80) (Jones et al., 2002), those with active neurological dysfunction that may affect cognitive processing (i.e., schizophrenia, Alzheimer's disease, Parkinson's disease, prior history of stroke, epilepsy, mental retardation and attention deficit-hyperactivity disorder) as well as those using antipsychotic and/or antiepileptic medications with known neurological/cognitive side effects were excluded. Individuals with previous brain surgery, serious CNS trauma as defined by a history of acquired sub- or epidural hematomas, or loss of consciousness for greater than 5 minutes were not included. Participants taking antidepressants were enrolled if they had been on stable treatment for more than 2 months and were not depressed according to the Center for Epidemiological Studies Depression Scale (CES-D) (Haringsma et al., 2004) or Profile of Moods Survey (POMS) (Shacham, 1983).

#### Physical

Participants with a body mass index (BMI kg/m^2^) (Keys et al., 1972) of less than 18 (normal) or greater than 35 (moderately obese) were excluded from the study in order to help control for the interaction of weight and alcohol metabolism (Sayon-Orea et al., 2011). Adults with diabetes (insulin-dependent) were also excluded, and individuals with high blood pressure were only included if they had been on stable treatment for at least 1 year (Hillbom et al., 2011). Other physical exclusions included visual acuity less than 20/40 (corrected), hearing loss, left-handed individuals, and positive pregnancy test results.

### Procedure

Potential participants completed a phone screen with questions about their alcohol, drug and medication use. A modified version of the SHORT AUDIT-C (Babor et al., 2001) was administered as part of the phone screen. This modified version included Questions #1 and #2 of the original version but excluded Question #3. This question was removed because it directly related to binge drinking, which was assessed elsewhere in the phone screen. The sum of Questions #1 and #2 were used as an initial measure of alcohol consumption. Participants with a score between 1 and 2 were conditionally recruited as light drinkers, and a score between 4 and 6 as moderate drinkers. See Supplemental Figure 1 for additional scoring information. Participants were also asked how long they had maintained their particular drinking pattern and how long they had been exposed to alcohol. All participants were asked whether or not they would agree to submit to an alcohol and drug screen at the beginning of each study visit.

Individuals that met phone screen criteria were asked to come to the laboratory for formal screening. Participants were given time to record 3 months of alcohol consumption using the TLFB. A 12 oz. (beer), 5 oz. (wine), and a 1.5 oz. (hard liquor) glass were placed on the participant's desk; they were told that the glasses represented standard drink sizes. Participants were asked to use these glasses as reference when filling out the TLFB. The following were also administered during the formal screening visit: mood (POMS) and depression questionnaires (CES-D), vision and hearing tests, medical history and Axis I disorder assessments (SCID), and a dementia screen (3MSE).

#### Testing visit one

Participants that passed formal screening criteria were officially enrolled into the study and asked to return on a separate day for 2.5 h of cognitive testing separated by two 15-minute breaks. The domains that were tested included attention, working memory, short-term memory, processing speed, planning, rule learning, and motor control. For a list of the tasks and behavioral measures used to assess performance see Table 1A.

**Table 1A:** **Tasks and measures used to assess domain-specific cognition during Testing Visit One**.

	**Measure**	**References**
**ATTENTION**		
Eriksen Flanker Test	Incongruent–Congruent Percent Accuracy	Eriksen and Eriksen, 1974
	Incongruent–Congruent Reaction Time	
**WORKING MEMORY**		
1Back Test	Percent Accuracy	Kirchner, 1958
	Reaction Time	
Delayed Match to Sample Test	Percent Accuracy	Sahakian et al., 1988
	Reaction Time	
**SHORT-TERM MEMORY**		
Spatial Recognition Test	Percent Accuracy	Owen et al., 1990
	Reaction Time	
Pattern Recognition Test	Percent Accuracy	Sahakian et al., 1990
	Reaction Time	
Spatial Span Test	Maximum Span Recalled	Milner, 1971; Owen et al., 1990
Hopkins Verbal Learning Test	Retention	Brandt, 1991; Benedict et al., 1998
**PROCESSING SPEED**		
Reaction Time Test	Reaction Time	www.cambridgecognition.com
Symbol Digit Modality Test	Number Correct	Smith, 1968, 1982
**PLANNING**		
Stockings of Cambridge Test	Number Answered in Minimum Moves	Robbins et al., 1998
Trails Making Test	Trials B–Trials A Completion Time	Reitan, 1958; Reitan and Wolfson, 1985
**RULE LEARNING**		
Intra-Extra Dimensional Set Shift Test	Total Number of Errors	Downes et al., 1989
**MOTOR CONTROL**		
Simple Reaction Time Test	Movement Time	www.cambridgecognition.com

#### Testing visit two

Measures of performance on cognitive tests are the primary outcomes of this study. However, approximately one year after beginning recruitment the study became affiliated with a larger NIAAA-funded project grant focused on interactions of stress and anxiety with alcohol consumption. It was at that time that measures of stress, anxiety, and impulsivity were added as secondary outcome measures and used to more thoroughly characterize older adult drinkers (Table 1B). Of the 13 light older drinkers that had already completed Testing Visit One, 10 returned to complete measures of stress, anxiety and impulsivity as part of Testing Visit Two. All 14 moderate older drinkers that already completed Testing Visit One were able to return and completed Testing Visit Two. The TLFB was re-administered upon their return to laboratory to ensure that light and moderate drinkers had not altered their drinking habits. No significant differences in the number of drinks per week were found in either light or moderate drinkers between Testing Visit One and Testing Visit Two [Light Old: *t*_(9)_ = −1.33, *p* = 0.22; Moderate Old: *t*_(13)_ = 1.45, *p* = 0.16]. Individuals recruited after the addition of Testing Visit Two (seven light and seven moderate older drinkers) completed the entire study within one month of enrollment.

**Table 1B:** **Questionnaires and measures used to assess stress, anxiety and impulsivity during Testing Visit Two**.

	**Measure**	**References**
**STRESS**		
Perceived Stress Scale	Total Score	Cohen et al., 1983
**ANXIETY**		
Geriatric Anxiety Scale	Total Score	Pachana et al., 2007
**IMPULSIVITY**		
Barrett Impulsivity Scale	Total Score	Patton et al., 1995
Lifetime History of Impulsive Behavior	Total Score	Schmidt et al., 2004; Coccaro and Schmidt-Kaplan, 2012
**IMPULSE CONTROL**		
Go No-Go Task	Cue Dependent Response Inhibition	Fillmore, 2003; Derefinko et al., 2008
	Cue Dependent Response Time	

### Statistical analysis

Multivariate analyses of covariance (MANCOVA) were used to assess the relationships between age (young, old) and alcohol consumption (light, moderate) with results from each cognitive test after controlling for four confounding variables: presence or absence of high blood pressure, BMI, total number of years consuming alcohol and the total number of years the individual maintained either a light or moderate alcohol consumption pattern. Additionally, sex differences were assessed and the drinking and age groups were collapsed across sex if found to be non-significant. Tests of normality were performed for each set of dependent variables (given the covariates) and any non-normally distributed variables were transformed appropriately (log transformed in our cases). Co-linearity and outlier assessments were also performed to ensure model validity. Step-down tests of the least square means were performed employing Tukey's adjustment for multiple comparisons if a significant main effect of alcohol, age or their interaction was found. All statistical analyses were performed using SAS software version 9.3.

## Results

### Demographics

Sample characteristics for both light and moderate older drinkers can be found in Table 2. Light older drinkers consumed 1.1 ± 0.7 drinks per week and moderate older drinkers consumed 11.8 ± 4.1 drinks per week. An expected significant difference in the number of drinks per week consumed by light and moderate older drinkers was observed [*t*_(39)_ = −11.6, *p* < 0.0001]. The two groups did not differ from each other in the total number of years they had maintained that consumption level [light: 21.0 ± 16.1 years, moderate: 16.1 ± 14.4 years; *t*_(39)_ = 1.0, *p* = 0.31] or the total number of years they had been consuming alcohol [light: 46.3 ± 10.0 years, moderate: 43.4 ± 11.5 years; *t*_(39)_ = 0.87, *p* = 0.39]. There were no statistical differences between light and moderate older drinkers in sex [light: 12 male, 8 female, moderate: 10 male, 11 female; χ^2^ = 0.63, *p* = 0.43], age [light: 71.1 ± 3.4 years old, moderate: 70.1 ± 4.2 years old; *t*_(39)_ = 0.80, *p* = 0.43], BMI [light: 27.4 ± 3.8, moderate: 26.3 ± 2.5; *t*_(39)_ = 1.12, *p* = 0.27], or years of education [light: 16.2 ± 3.0 years, moderate: 16.5 ± 2.1 years; *t*_(39)_ = −0.40, *p* = 0.69]. Older drinkers scored similarly on depression [CES-D, light: 4.5 ± 3.6, moderate: 4.3 ± 4.9; *t*_(39)_ = 0.12, *p* = 0.90] and mood screens [POMS, light: −3.4 ± 11.0, moderate: −1.6 ± 13.8; *t*_(39)_ = −0.46, *p* = 0.65]. Older drinkers also performed similarly on the dementia screen [3MSE, light: 97.1 ± 3.4, moderate: 97.8 ± 3.0; *t*_(39)_ = −0.71, *p* = 0.48]. No significant difference in the proportion of people on a stable regimen of prescription medication was found (light: 16/20, moderate: 17/21, χ^2^ = 0.01, *p* = 0.94). More specifically, no differences in the proportion of people on medication to treat high blood pressure (light: 7/20, moderate: 10/21, χ^2^ = 1.33, *p* = 0.25), high cholesterol (light: 4/20, moderate: 3/21, χ^2^ = 0.24, *p* = 0.67), Type II diabetes (light: 2/20, moderate: 1/21, χ^2^ = 0.42, *p* = 0.52) or depression were found (light:1/20, moderate: 5/21, χ^2^ = 2.9011, *p* = 0.09). Other commonly cited conditions that required prescription medication in both light and moderate older drinkers included hypothyroidism, arthritis, osteoporosis, and heartburn. In the light older drinker group, only one individual smoked (less than thirty cigarettes per day) and there were no ex-smokers. In the moderate older drinker group, no one actively smoked cigarettes; one individual was an ex-smoker.

**Table 2:** **Light and moderate older adult drinker sample characteristics**.

**Mean ± SD (Min–Max)**	**Sex**	**Age**	**BMI**	**Education**	Drinks week^*^	**Pattern maintenance**	**Lifetime drinking**
**Older Adults**	**M/F**	**Years**		**Years**		**Years**	**Years**
Light	12|8	71.1±3.4	27.4±3.8	16.2±3.0	1.1±0.7	21.0±16.1	46.3±10.0
(*n* = 20)		(66–75)	(22–35)	(12–25)	(0.1–2.8)	(3–54)	(15–60)
Moderate	10|11	70.1±4.2	26.3±2.5	16.5±2.1	11.8±4.1	16.1±14.4	43.4±11.5
(*n* = 21)		(63–78)	(23–31)	(12–20)	(7–19)	(4–51)	(10–55)

*Drinks per week were calculated using the self-report number of drinks in the past 3 months using the Time Line Follow Back Questionnaire. BMI = body mass index. BMI, whether or not an individual had high blood pressure, the number of years an individual maintained either a light or moderate drinking pattern, and the total number of years an individual drank alcohol were used as covariates during statistical analysis. Seven light and 10 moderate older drinkers were on a stable medication regiment to treat high blood pressure*.

* *p < 0.05 for null hypothesis of no difference between light and moderate older adult drinkers*.

For comparative purposes, sample characteristics for young adult drinkers can be found in Table 3. Light younger drinkers (27.2 ± 3.3 years old) consumed 1.6 ± 0.6 drinks per week and moderate younger drinkers (27.5 ± 3.7 years old) consumed 10.8 ± 2.6 drinks per week. These consumption patterns were statistically similar to the consumption patterns observed in light [*t*_(29)_ = 1.89, *p* = 0.07] and moderate [*t*_(30)_ = −0.70, *p* = 0.50] older adult drinker counterparts. The most notable medication taken by younger adult drinkers was birth control and none of the younger adult drinkers smoked cigarettes.

**Table 3:** **Light and moderate younger adult drinker sample characteristics**.

**Mean ± SD (Min–Max)**	**Sex**	**Age**	**BMI**	**Education**	Drinks week^*^	**Pattern maintenance**	**Lifetime drinking**
**Younger Adults**	**M/F**	**Years**		**Years**		**Years**	**Years**
Light	5|6	27.2±3.3	22.4±3.5	19.4±2.3	1.6±0.6	4.6±2.2	7.5±2.7
(*n* = 11)		(24–35)	(18–29)	(15–22)	(0.4–2)	(3–11)	(3–12)
Moderate	5|6	27.5±3.7	23.9±3.5	19.1±2.3	10.8±2.6	5.3±1.7	9.2±4.1
(*n* = 11)		(24–35)	(20–30)	(16–24)	(8–17)	(3–9)	(5–18)

*Drinks per week were calculated using the self-report number of drinks in the past 3 months using the Time Line Follow Back Questionnaire. BMI = body mass index. BMI, whether or not an individual had high blood pressure, the number of years an individual maintained either a light or moderate drinking pattern, and the total number of years an individual drank alcohol were used as covariates during statistical analysis. Neither light nor moderate younger drinkers were on a stable medication regiment to treat high blood pressure*.

* *p < 0.05 for null hypothesis of no difference between light and moderate younger adult drinkers*.

### Cognitive task performance

#### Effects of age

Age ^*^ alcohol consumption MANCOVA results are presented in Table 4. As expected, significant effects of age showed that younger adults outperformed older adults in several cognitive domains, including executive attention [*F*_(54)_ = 7.25, *p* < 0.01], working-memory [F_(52)_ = 7.29, *p* < 0.0001], short-term memory [*F*_(50)_ = 4.58, *p* < 0.0001], processing speed [*F*_(54)_ = 6.09, *p* = 0.01], and rule learning [*F*_(52)_ = 9.73, *p* < 0. 01]. Younger adults trended toward faster movement times than older adults in the motor control domain [*F*_(55)_ = 3.13, *p* = 0.08] but did similarly to older adults in the planning domain [*F*_(54)_ = 1.31, *p* = 0.28]. See Table 5 for the estimated performance means and 95% confidence intervals for both younger and older drinkers.

**Table 4:** Results of consumption level (low, moderate) * age group (young, old) MANCOVA analyses of cognitive performance.

	**Age**	**Drink**	Drink * Age
Executive Attention	*F*_(54)_ = 7.25	*F*_(54)_ = 1.99	*F*_(54)_ = 2.59
	*p* < 0.01^*^	*p* = 0.15	*p* = 0.08
Working Memory	*F*_(52)_ = 7.29	*F*_(52)_ = 2.54	*F*_(52)_ = 1.84
	*p* < 0.0001^*^	*p* = 0.05^*^	*p* = 0.14
Other executive functions			
Short-term memory	*F*_(50)_ = 4.58	*F*_(50)_ = 1.05	*F*_(50)_ = 1.00
	*p* < 0.0001^*^	*p* = 0.40	*p* = 0.44
Processing speed	*F*_(54)_ = 6.09	*F*_(54)_ = 0.11	*F*_(54)_ = 0.08
	*p* < 0.01^*^	*p* = 0.90	*p* = 0.93
Planning	*F*_(54)_ = 1.31	*F*_(54)_ = 1.51	*F*_(54)_ = 0.93
	*p* = 0.28	*p* = 0.23	*p* = 0.40
Rule learning	*F*_(52)_ = 9.73	*F*_(52)_ = 0.92	*F*_(52)_ = 4.50
	*p* < 0.01^*^	*p* = 0.34	*p* = 0.04^*^
Motor Control	*F*_(55)_ = 3.13	*F*_(55)_ = 0.22	*F*_(55)_ = 0.69
	*p* = 0.08	*p* = 0.64	*p* = 0.41

**Table 5:** **Cognitive test performance in younger and older adult drinkers**.

		**Younger drinkers**		**Older drinkers**	
		**Estimated mean**	**95% CI**	**Estimated mean**	**95% CI**
**ATTENTION**					
Eriksen Flanker Test	Incongruent–Congruent	4.08%	−9.80–17.95%	−16.60%	−24.81–(−8.39)%
	Percent Accuracy^*^				
	Incongruent–Congruent	43.25 ms	2.24–84.25 ms	147.62 ms	123.35–171.88 ms
	Reaction Time^*^				
**WORKING MEMORY**					
1Back Test	Percent Accuracy	98.31%	85.95–110.66%	89.61%	82.30–96.92%
	Reaction Time	777.07 ms	667.20–886.93 ms	857.28 ms	729.26–922.30 ms
Delayed Match to Sample Test	Percent Accuracy^≈^	86.80%	78.83–94.77%	76.50%	71.79–81.22%
	Reaction Time^*^	1637.65 ms	933.74–2341.56 ms	4221.14 ms	3804.54–4637.73 ms
**SHORT-TERM MEMORY**					
Spatial Recognition Test	Percent Accuracy^*^	81.55%	70.02–94.98%	60.50%	55.28–66.22%
	Reaction Time^*^	1504.97 ms	1205.32–1879.11 ms	2600.83 ms	2280.58–2966.06 ms
Pattern Recognition Test	Percent Accuracy	93.30%	87.61–99.36%	91.37%	88.03–94.83%
	Reaction Time^*^	1369.64 ms	1182.64–1586.22 ms	2276.83 ms	2087.37–2483.49 ms
Spatial Span Test	Maximum Span Recalled^*^	6.59	5.60–7.75	5.23	4.75–5.76
Hopkins Verbal Learning Test	Retention	96.82%	77.77–120.53%	95.17%	83.59–108.34%
**PROCESSING SPEED**					
Reaction Time Test	Reaction Time^*^	269.24 ms	233.84–304.64 ms	323.19 ms	302.24–344.14 ms
Symbol Digit Modality Test	Number Correct^*^	60.06	52.87–67.25	41.96	37.70–46.21
**PLANNING**					
Stockings of Cambridge Test	Number Answered in Minimum Moves^≈^	9.45	8.02–10.88	7.63	6.79–8.48
Trails Making Test	Trials B−Trials A Completion Time	39.75 ms	7.84–71.66 ms	36.30 ms	17.41–55.18 ms
**RULE LEARNING**					
Intra-Extra Dimensional Set Shift Test	Total Number of Errors^*^	10.02	6.62–15.17	22.18	17.36–28.35
**MOTOR CONTROL**					
Simple Reaction Time Test^*^	Movement Time	363.62 ms	302.50–437.10 ms	499.91 ms	448.32–557.44 ms

Estimated performance means and 95% confidence intervals associated with each cognitive test collapsed across age.

* *Denotes a statistically significant finding of p ≤ 0.05, and ^≈^denotes a nearly significant finding of p = 0.08*.

#### Effects of alcohol consumption

A modest effect of alcohol consumption was found in the working memory domain [*F*_(52)_ = 2.54, *p* = 0.05]. No differences in 1Back Task percent accuracy were found [Light = 95.26%, 89.27–101.25%; Moderate = 92.66%, 86.75–98.58%; *t*_(55)_ = 0.68, *p* = 0.50]. However, moderate drinkers responded faster during the 1Back Task [Light = 861.09 ms, 807.83–914.35 ms; Moderate = 773.26 ms, 720.65–825.87 ms; *t*_(55)_ = 2.57, *p* = 0.01]. This finding was driven by a nearly significant difference between the reaction times of younger drinkers [Light Young = 844.19 ms, 717.83–970.55 ms; Moderate Young = 709.94 ms, 590.47–829.41 ms; *t*_(55)_ = 2.44, *p* = 0.08] but not older drinkers [Light Old = 877.98 ms, 797.84–958.14 ms; Moderate Old = 836.58 ms, 762.57–910.58 ms; *t*_(55)_ = 1.0, *p* = 0.75]. Moderate drinkers performed better on the Delayed Match to Sample Task [Light = 79.15%, 75.29–83.02%; Moderate = 84.15%, 80.33–87.96%; *t*_(55)_ = −2.02, *p* = 0.05]. This finding was driven by a nearly significant difference between the percent accuracies of older adults [Light Old = 72.20%, 66.89–78.51%; Moderate Old = 80.31%, 74.94–85.67%; *t*_(55)_ = −2.53, *p* = 0.07] but not younger adults [Light Young = 85.61%, 76.44–94.77%; Moderate Young = 87.99%, 79.33–96.66%; *t*_(55)_ = −0.60, *p* = 0.93]. No difference between light and moderate drinker Delayed Match to Sample Task reaction times was found [Light = 2844.25 ms, 2503.01–3185.49 ms; Moderate = 3014.53 ms, 2677.48–3351.59; *t*_(55)_ = −0.78, *p* = 0.44]. See Table 6 for the estimated performance means and 95% confidence intervals for both light and moderate drinkers.

**Table 6:** **Cognitive test performance in light and moderate adult drinkers**.

		**Light drinkers**		**Moderate drinkers**	
		**Estimated mean**	**95% CI**	**Estimated mean**	**95% CI**
**ATTENTION**					
Eriksen Flanker Test	Incongruent-Congruent	−4.74%	−11.46–1.99%	−7.79%	−14.43–(−1.14)%
	Percent Accuracy				
	Incongruent-Congruent	104.53 ms	84.65–124.41 ms	86.33 ms	66.70–105.97 ms
	Reaction Time				
**WORKING MEMORY**					
1Back Test	Percent Accuracy	95.26%	89.27–101.25%	92.66%	86.75–98.58%
	Reaction Time^*^	861.09 ms	807.83–914.35 ms	773.26 ms	720.65–825.87 ms
Delayed Match to Sample Test	Percent Accuracy^*^	79.15%	75.29–83.02%	84.15%	80.33–87.96%
	Reaction Time	2844.25 ms	2503.01–3185.49 ms	3014.53 ms	2677.48–3351.59 ms
**SHORT-TERM MEMORY**					
Spatial Recognition Test	Percent Accuracy	71.58%	66.48–77.07%	68.93%	64.07–74.15%
	Reaction Time	2062.49 ms	1852.02–2296.87 ms	1897.79 ms	1706.38–2110.66 ms
Pattern Recognition Test	Percent Accuracy	91.87%	89.11–94.71%	92.79%	90.04–95.63%
	Reaction Time	1829.76 ms	1704.07–1964.73 ms	1704.29 ms	1588.61–1828.40 ms
Spatial Span Test	Maximum Span Recalled	5.78	5.34–6.25	5.96	5.52–6.45
Hopkins Verbal Learning Test	Retention	95.10%	85.52–105.75%	96.88%	87.24–107.60%
**PROCESSING SPEED**					
Reaction Time Test	Reaction Time	293.09 ms	275.93–310.25 ms	299.34 ms	282.39–316.29 ms
Symbol Digit Modality Test	Number Correct	49.99	46.50–53.47	52.03	48.49–55.47
**PLANNING**					
Stockings of Cambridge Test	Number Answered in Minimum Moves	8.59	7.90–9.28	8.49	7.81–9.18
Trails Making Test	Trials B-Trials A Completion Time	41.85 ms	26.38–57.32 ms	34.20 ms	18.92–49.47 ms
**RULE LEARNING**					
Intra-Extra Dimensional Set Shift Test	Total Number of Errors	15.50	12.68–18.95	14.34	11.76–17.48
**MOTOR CONTROL**					
Simple Reaction Time Test	Movement Time	425.37 ms	389.06–465.06 ms	427.35 ms	391.30–466.71 ms

*Estimated performance means and 95% confidence intervals associated with each cognitive test collapsed across consumption level*.

* *Denotes a statistically significant finding of p ≤ 0.05*.

#### Interactive effects of age and alcohol consumption

A modest age ^*^ alcohol consumption interaction effect was found in the rule learning domain [*F*_(52)_ = 4.50, *p* = 0.04]. Further analysis showed that light old drinkers tended to make the most errors during the Intra-Extra Dimensional Set Shift Task. More specifically, light old drinkers (26.58 errors, 19.49–35.87 errors) were found to make significantly more errors than both light young [9.03 errors, 5.64–14.56 errors; *t*_(55)_ = −3.12, *p* = 0.02] and moderate young drinkers [11.02 errors, 7.03–17.46 errors; *t*_(55)_ = −2.65, *p* = 0.05]. Although the number of errors did not significantly differ between older drinkers [*t*_(55)_ = 2.28, *p* = 0.11], moderate old drinkers [18.54 errors, 14.01–24.53 errors] performed just as well as light young [*t*_(55)_ = −2.17, *p* = 0.15] and moderate young drinkers [*t*_(55)_ = −1.63, *p* = 0.37].

A nearly significant age ^*^ alcohol consumption interaction effect was found in the executive attention domain (*F*_(54)_ = 2.59, *p* = 0.08). Light old drinkers tended to have the lowest percent accuracy on the Eriksen Flanker Task (Light Young = 10.51%, −5.45–26.46%; Moderate Young = −2.35%, −17.44–12.73%; Light Old = −19.98%, −30.10 to 9.86%; Moderate Old = −13.22%, −22.56 to −3.87%). In addition to this, light old drinkers tended to exhibit the slowest reaction times (Light Young = 43.75 ms, −3.41 to 90.91 ms; Moderate Young = 42.74 ms, −1.85–87.33 ms; Light Old = 165.31 ms, 135.40–195.22 ms; Moderate Old = 129.93 ms, 102.30–157.55 ms).

No significant age ^*^ alcohol consumption interaction effects were found in working memory [*F*_(52)_ = 1.84, *p* = 0.14], short-term memory [*F*_(50)_ = 1.00, *p* = 0.44], processing speed [*F*_(54)_ = 0.08, *p* = 0.93], planning [*F*_(54)_ = 0.93, *p* = 0.40] or motor control [*F*_(55)_ = 0.69, *p* = 0.41] domains. See Table 7 for the estimated performance means and 95% confidence intervals for light and moderate older drinkers and Table 8 for light and moderate younger drinkers.

**Table 7:** **Cognitive test performance in light and moderate older adult drinkers**.

		**Light older drinkers**		**Moderate older drinkers**	
		**Estimated mean**	**95% CI**	**Estimated mean**	**95% CI**
**ATTENTION**					
Eriksen Flanker Test	Incongruent–Congruent	−19.98%	−30.10–(−9.86)%	−13.22%	−22.56–(−3.87)%
	Percent Accuracy				
	Incongruent–Congruent	165.31 ms	135.40–195.22 ms	129.93 ms	102.30–157.55 ms
	Reaction Time				
**WORKING MEMORY**					
1Back Test	Percent Accuracy	92.85%	83.84–101.86%	86.38%	78.05–94.70%
	Reaction Time	877.98 ms	797.84–958.12 ms	836.58 ms	762.57–910.58 ms
Delayed Match to Sample Test	Percent Accuracy	72.70%	66.89–78.51%	80.31%	74.94–85.67%
	Reaction Time	4198.06 ms	3684.59–4711.53 ms	4244.20 ms	3770.06–4718.36 ms
**SHORT-TERM MEMORY**					
Spatial Recognition Test	Percent Accuracy	58.95%	52.75–65.89%	62.10%	56.03–68.81%
	Reaction Time	2734.62 ms	2325.73–3215.40 ms	2473.59 ms	2129.98–2872.62 ms
Pattern Recognition Test	Percent Accuracy	90.80%	86.73–95.07%	91.94%	88.12–95.92%
	Reaction Time	2310.04 ms	2075.45–2571.14 ms	2244.11 ms	2032.82–2477.36 ms
Spatial Span Test	Maximum Span Recalled	5.16	4.58–5.81	5.31	4.75–5.92
Hopkins Verbal Learning Test	Retention	95.17%	81.12–111.67%	95.16%	82.10–110.29%
**PROCESSING SPEED**					
Reaction Time Test	Reaction Time	319.72 ms	293.89–345.54 ms	326.66 ms	302.81–350.50 ms
Symbol Digit Modality Test	Number Correct	40.50	35.26–45.74	43.42	38.57–48.26
**PLANNING**					
Stockings of Cambridge Test	Number Answered in Minimum Moves	7.54	6.50–8.59	7.72	6.76–8.69
Trails Making Test	Trials B–Trials A Completion Time	39.49 ms	16.21–62.77 ms	33.10 ms	11.61–54.59 ms
**RULE LEARNING**					
Intra-Extra Dimensional Set Shift Test	Total Number of Errors	26.50	19.59–35.86	18.56	14.04–24.54
**MOTOR CONTROL**					
Simple Reaction Time Test	Movement Time	486.81 ms	425.66–556.76 ms	513.36 ms	453.51–581.11 ms

**Table 8:** **Cognitive test performance in light and moderate young adult drinkers**.

		**Light younger drinkers**		**Moderate younger drinkers**	
		**Estimated mean**	**95% CI**	**Estimated mean**	**95% CI**
**ATTENTION**					
Eriksen Flanker Test	Incongruent-Congruent	10.51%	−5.45–26.46%	−2.35%	−17.44–12.73%
	Percent Accuracy				
	Incongruent–Congruent	43.75 ms	−3.41–90.91 ms	42.74 ms	−1.85–87.33 ms
	Reaction Time				
**WORKING MEMORY**					
1Back Test	Percent Accuracy	97.67%	83.46–111.88%	98.95%	85.52–112.38%
	Reaction Time^≈^	844.19 ms	717.83–970.55 ms	709.94 ms	590.47–829.41 ms
Delayed Match to Sample Test	Percent Accuracy	85.61%	76.44–94.77%	87.99%	79.33–96.66%
	Reaction Time	1490.44 ms	680.86–2300.02 ms	1784.85 ms	1019.44–2550.27 ms
**SHORT-TERM MEMORY**					
Spatial Recognition Test	Percent Accuracy	86.92%	72.94–103.58%	76.51%	64.82–90.31%
	Reaction Time	1555.55 ms	1204.99–2008.10 ms	1456.02 ms	1143.72–1853.61 ms
Pattern Recognition Test	Percent Accuracy	92.94%	86.45–99.92%	93.66%	87.46–100.29%
	Reaction Time	1449.34 ms	1224.18–1715.92 ms	1294.33 ms	1103.36–1518.34 ms
Spatial Span Test	Maximum Span Recalled	6.47	5.37–7.81	6.70	5.61–8.00
Hopkins Verbal Learning Test	Retention	95.02%	73.86–122.25%	98.64%	77.74–125.18%
**PROCESSING SPEED**					
Reaction Time Test	Reaction Time	266.46 ms	225.75–307.17 ms	272.03 ms	233.53–310.17 ms
Symbol Digit Modality Test	Number Correct	59.47	51.20–67.74	60.64	52.82–68.46
**PLANNING**					
Stockings of Cambridge Test	Number Answered in Minimum Moves	9.64	8.00–11.28	9.26	7.71–10.82
Trails Making Test	Trials B–Trials A Completion Time	44.21 ms	7.51–80.91 ms	35.29 ms	0.59–69.99 ms
**RULE LEARNING**					
Intra–Extra Dimensional Set Shift Test	Total Number of Errors	9.07	5.63–14.61	11.07	7.05–17.38
**MOTOR CONTROL**					
Simple Reaction Time Test	Movement Time	371.67 ms	300.77–459.29 ms	355.74 ms	291.22-434.56 ms

≈ *Denotes a nearly significant finding of p = 0.08*.

### Stress, anxiety, and impulsivity

Light and moderate older drinkers did not differ from each other in measures known to influence cognition and overall alcohol consumption. Neither perceived stress nor anxiety showed a significant effect of alcohol consumption [Perceived Stress Score: *F*_(32)_ = 2.02, *p* = 0.32 and Geriatric Anxiety Scale: *F*_(31)_ = 0.02, 0.89]. No significant effects of alcohol consumption were found in either personality measures of impulsivity [Barrett Impulsivity Scale: *F*_(30)_ = 2.18, *p* = 0.11; Lifetime History of Impulsive Behavior Inventory: *F*_(31)_ = 0.07, *p* = 0.79] or the behavioral measure of impulse control [Go No-Go Task: *F*_(31)_ = 0.53, *p* = 0.60]. See Table 9 for the estimated performance means and 95% confidence intervals for both light older and moderate older drinkers.

**Table 9:** **Assessment of stress, anxiety and impulsivity in older drinkers**.

		**Light older drinkers**		**Moderate older drinkers**	
		**Estimated mean**	**95% CI**	**Estimated mean**	**95% CI**
**STRESS**					
Perceived Stress Scale	Total Score	2.24	1.92–2.56	2.02	1.73–2.31
**ANXIETY**					
Geriatric Anxiety Scale	Total Score	2.20	1.78–2.62	2.29	1.91–2.66
**IMPULSIVITY**					
Barrett Impulsivity Scale	Total Score	52.17	49.06–55.28	54.15	51.37–56.93
Lifetime History of Impulsive Behavior	Total Score	41.04	32.91–49.17	43.35	36.07–50.62
**IMPULSE CONTROL**					
Go No-Go Task	Cue Dependent Response Inhibition	0.01	−0.01–0.03	0.03	0.01–0.05
	Cue Dependent Response Time	12.97 ms	3.34–22.61 ms	10.75 ms	2.14–19.36 ms

## Discussion

This study was specifically designed to measure the effect of long-term moderate alcohol consumption on cognitive health in older adult drinkers. Expected age-related deficits in cognitive performance were observed but no evidence was found to suggest that long-term moderate alcohol consumption in older adults exacerbated age-related cognitive decline.

Interestingly, moderate older drinkers outperformed light older drinkers on the Delayed Match to Sample Task. This finding suggests that long-term moderate alcohol consumption in older age may be associated with select benefits to cognition, and in particular the short-term memory of learned relationships. However, when interpreting this finding it is important to make note of studies that show parameter-dependent performance in alcoholics on this task (Oscarberman and Bonner, 1985; Holden et al., 2012), and remember that potential benefits are likely to be highly specific and thus of have lower ecological relevance. This study also found that moderate younger drinkers responded to 1Back Task trials faster than light younger drinkers despite showing similar performance. This finding helps to support the idea that alcohol exposure can affect speed and accuracy on working memory tasks in different manners (Schweizer and Vogel-Sprott, 2008; Wilcox et al., 2014). It may also be indicative of a potential difference between younger drinkers in the interaction between impulsivity and different components of a working memory task. To help support this are previous alcohol research studies showing direct relationships between measures of impulsivity and working memory capacity (Gunn and Finn, 2013; Noel et al., 2013; Ellingson et al., 2014). Although studies were based on heavy alcohol drinkers, our findings may point to the sensitivity of these relationships to alcohol exposure at lower quantities in younger individuals.

Accounts detailing the effects of long-term moderate alcohol consumption on cognitive health were first published approximately 30 years ago (Goodwin et al., 1987; Herbert et al., 1993; Hendrie et al., 1996; Anstey et al., 2009). These and subsequent studies have been predominately epidemiologic in nature and dominated by analyses that use secondary outcome measures from large-scale clinical trials, like the Framingham Heart Study (Elias et al., 1999), the Seattle Longitudinal Study (Zanjani et al., 2013) and the Women's Health Initiative (Espeland et al., 2005, 2006). Although these analyses benefit from large sample sizes and longitudinal queries have been able to explore causal relationships between alcohol consumption and cognition (Edelstein et al., 1998; Virtaa et al., 2010), the parent clinical trials were not specifically designed to measure the effect of moderate alcohol consumption on age-related cognitive decline as the primary outcome. Consequently, there is little consensus in the literature, and the effect of moderate alcohol consumption on cognitive health in older age is unclear. To illustrate this point, studies have shown a negative effect (Dufouil et al., 2000; Zhou et al., 2003), a positive effect (Arntzen et al., 2010; Zanjani et al., 2013), as well as no consistent effect (Almeida et al., 2014; Sabia et al., 2014).

Strengths associated with this report include the definition of moderate alcohol consumption. In previous research, the definition of moderate has been broad and has ranged from 5 to 60 g of alcohol per day (approximately 1 to 6 drinks per day) depending on the tool used for assessment. In this study, the definition for moderate alcohol consumption was based on alcohol use trends observed in older American drinkers (NIAAA, 1998, 2006; Fryar et al., 2006) and helped make study criteria more generalizable. Another important addition to literature provided by this study was the attention paid to the quantity and frequency of alcohol consumption. Although the aggregate weekly intake volume for moderate consumption (approximately 91–273 g of alcohol) may seem lenient, the pattern with which it was consumed was considered in detail. For example, moderate consumption was not allowed to exceed 3 drinks (approximately 39 g of alcohol) per day and individuals that reported binge-drinking habits were not included in the study. Participants were also explicitly asked about their lifetime alcohol consumption habits, and were balanced in terms of the number of years they had maintained their consumption level as well as the total number of years they were exposed to alcohol. Individuals were thoroughly questioned about their drinking habits using a series of validated questionnaires to ensure those with present or past problematic drinking patterns were excluded. Furthermore, light drinkers were used as the control group. This bolstered ecological validity given recent census data and avoided any differences between non-drinkers and drinkers that are difficult to account for during analysis (e.g., religion or distaste for alcohol) (Green and Polen, 2001; Rehm et al., 2008). In addition, the study population was specifically restricted to healthy, active and community dwelling adults. Older adults who fulfilled these criteria but who were also on a stable medication regimen were also enrolled. This helped to make findings generalizable to a growing number of older American adults, who are living longer and healthier lives thanks to medications designed to stabilize conditions like high blood pressure, depression, and hypothyroidism. The extensive cognitive battery used in this study adds to current research that has predominately used global assessments of mild cognitive impairment and dementia (Dent et al., 1997; Dufouil et al., 2000; Bond et al., 2001, 2005; Chan et al., 2010). The assessments used in this study are validated measures of specific cognitive domains (i.e., short term memory vs. working memory) and have been widely used in aging and substance use literature. To our knowledge, this is the first study specifically designed to measure an extensive list of cognitive abilities in older social drinkers. Analyses included cognitive aspects but also explored stress, anxiety and impulsivity, which are directly relevant to the effects of alcohol on both physical and cognitive health.

Research limitations include the TLFB method used to quantify alcohol consumption in this study. Although underrepresentation is a common concern with self-report alcohol consumption questionnaires, it should be noted that this phenomenon is more common to populations that consume alcohol in larger quantities than those discussed in this study (Stockwell et al., 2004). Limitations to the study also include a relatively small sample size when compared to previous research based on large-scale clinical trials. Despite this, confidence in our findings was strengthened by the fact that the presented estimated performance means are similar to those previously reported in healthy older adults (Sahakian et al., 1988, 1990; Downes et al., 1989; Owen et al., 1990; Robbins et al., 1998; Shapiro et al., 1999; de Jager et al., 2002; Hogervorst et al., 2002; De Luca et al., 2003; Hoyer et al., 2004; Tombaugh, 2004; Van Gerven et al., 2008).

In summary, this study showed that long-term moderate alcohol consumption in older age is neither harmful nor beneficial to overall cognitive health. Given the fact that previous findings in this field have been inconsistent (i.e., both positive and negative) the finding of this tailored experiment likely represents the most probable effect of long-term light to moderate alcohol consumption on cognitive performance in older adults: little to none. It is important to note, of course, that this finding is specific to healthy, community-dwelling and social drinkers. Findings should not be generalized to individuals with other consumption patterns, like heavy drinkers, binge drinkers and ex-drinkers.

### Conflict of interest statement

The authors declare that the research was conducted in the absence of any commercial or financial relationships that could be construed as a potential conflict of interest.
