# Supplementary material for: Long-term moderate alcohol consumption does not exacerbate age-related cognitive decline in healthy, community-dwelling older adults
Source: Front Aging Neurosci. 2015 Jan 5;6:341. doi: 10.3389/fnagi.2014.00341 (PMC4283638; doi:10.3389/fnagi.2014.00341)
Supplement: Supplementary file 1 [file Table1.DOCX]

1. **How often do you have a drink containing alcohol?**
   - Never (0 points)
   - Monthly or less (1 point)
   - 2-4 times a month (2 points)
   - 2-3 times a week (3 points)
   - 4 or more times a week (4 points)
2. **How many standard drinks containing alcohol do you have on a typical day?**
   - 1 or 2 (0 points)
   - 3 or 4 (1 point)
   - 5 or 6 (2 points)
   - 7 to 8 (3 points)
   - 10 or more (4 points)

**Supplemental Figure 1: Questions and scoring used to identify light and moderate drinkers during the phone screen**

A modified version of the SHORT Alcohol Use Disorders Identifications Test – C was used as an initial measure of alcohol consumption. Participants with a total score between 1 and 2 were conditionally recruited as light drinkers, and a score between 4 and 6 as moderate drinkers.
